# Bovine Pestivirus Heterogeneity and Its Potential Impact on Vaccination and Diagnosis

**DOI:** 10.3390/v12101134

**Published:** 2020-10-06

**Authors:** Victor Riitho, Rebecca Strong, Magdalena Larska, Simon P. Graham, Falko Steinbach

**Affiliations:** 1 Virology Department, Animal and Plant Health Agency, APHA-Weybridge, Woodham Lane, New Haw, Addlestone KT15 3NB, UK; vriitho@gmail.com (V.R.); Rebecca.Strong@apha.gov.uk (R.S.); 2 Department of Virology, National Veterinary Research Institute, Al. Partyzantów 57, 24-100 Puławy, Poland; maglar7@wp.pl; 3 The Pirbright Institute, Ash Road, Pirbright GU24 0NF, UK; simon.graham@pirbright.ac.uk; 4 School of Veterinary Medicine, University of Surrey, Guilford GU2 7XH, UK

**Keywords:** bovine pestiviruses, bovine viral diarrhoea, vaccination, control, diagnosis, antigenic cross-reactivity

## Abstract

Bovine Pestiviruses A and B, formerly known as bovine viral diarrhoea viruses (BVDV)-1 and 2, respectively, are important pathogens of cattle worldwide, responsible for significant economic losses. Bovine viral diarrhoea control programmes are in effect in several high-income countries but less so in low- and middle-income countries where bovine pestiviruses are not considered in disease control programmes. However, bovine pestiviruses are genetically and antigenically diverse, which affects the efficiency of the control programmes. The emergence of atypical ruminant pestiviruses (Pestivirus H or BVDV-3) from various parts of the world and the detection of Pestivirus D (border disease virus) in cattle highlights the challenge that pestiviruses continue to pose to control measures including the development of vaccines with improved cross-protective potential and enhanced diagnostics. This review examines the effect of bovine pestivirus diversity and emergence of atypical pestiviruses in disease control by vaccination and diagnosis.

## 1. Bovine Pestiviruses

The Pestivirus genus within the family Flaviviridae of single stranded positive sense RNA viruses comprises eleven recognized species, Pestivirus A-K [1]. The previously recognised species included bovine viral diarrhoea virus 1 (BVDV-1, now known as Pestivirus A), BVDV-2 (Pestivirus B), classical swine fever virus (CSFV, Pestivirus C) and border disease virus (BDV, Pestivirus D). In addition, a further 7 other species were designated as Pestivirus E-K: Pestivirus E (pronghorn antelope virus), Pestivirus F (porcine Pestivirus), Pestivirus G (giraffe Pestivirus), Pestivirus H (Hobi-like Pestivirus, atypical ruminant Pestivirus, also known as BVDV-3), Pestivirus I (Aydin-like Pestivirus, sheep Pestivirus), Pestivirus J (rat Pestivirus) and Pestivirus K (atypical porcine Pestivirus). The reclassification of species names is relatively new, and still not widely used, and thus for ease of comparing the literature, both nomenclatures will be used here throughout.

The classification of pestiviruses is based on genetic and antigenic relatedness as well as the host of origin [2]. Genetic likeness of pestiviruses has been shown to be consistent with antigenic relatedness as defined by binding assays with monoclonal antibodies (mAbs) or serum cross-neutralisation relative to the type virus of a particular species [3]. Pestiviruses differ in their host tropism with Pestivirus A, B and H mainly found in Bovidae or material thereof, hence the original naming as BVDV-1, -2 and -3. Phylogenetic analysis has identified 21 Pestivirus A subtypes (BVDV-1a-u) and 4 Pestivirus B subtypes (BVDV-2a-d) [4]. Pestiviruses H (BVDV-3), formerly referred to as atypical bovine pestiviruses, are a species with similar variability, but no defined subtypes as of yet [5]. Bovine pestiviruses can also infect other domestic livestock species such as sheep, goats and pigs [6]. Conversely, publications have demonstrated the infection of cattle with Pestivirus D (border disease virus) normally associated with the infection of small ruminants, including the ability to establish a persistent infection in bulls [7,8,9]. There is a need to understand the role of heterologous hosts in the transmission, spill over and emergence of bovine pestiviruses [10]. 

Pestivirus H (BVDV-3) represents a group of atypical ruminant pestiviruses that were first detected in commercial foetal bovine serum (FBS), originating from South America [11], Southeast Asia [12] or with unknown origin [13]. Other viruses have also been isolated from aborted bovine foetuses [14] and from buffalo in Brazil [15]. It is not clear whether cattle or other bovids are the natural reservoir/host of atypical ruminant pestiviruses. More recently, a Pestivirus H strain has been associated with a severe respiratory disease outbreak and abortions in multiparous cows in Italy [16]. Accordingly, there is evidence suggesting that Pestivirus H is spreading in cattle in South America [14], Southeast Asia [17] and Europe [16]. The extent to which they are present in the cattle population worldwide needs to be further assessed since the genetic and antigenic diversity between bovine pestiviruses poses a significant challenge in BVD diagnosis and vaccination [18].

## 2. Impact and Control of Bovine Pestiviruses

Bovine pestiviruses are an important group of pathogens that cause significant economic losses to the cattle industry worldwide [19]. BVD is well recognised as an economic factor of cattle production in the western world but less so in the developing world including emerging economies, such as Brazil, where it is not yet considered in disease control programmes. A meta-analysis of bovine pestivirus prevalence in 325 studies across 73 countries showed global prevalence with significantly higher prevalence in countries without BVDV control programmes [20]. The prevalence and impact of Pestivirus H is yet to be fully considered in such studies.

As a result of their economic impact, significant efforts are being made to prevent and control bovine pestiviruses in many developed countries, particularly in Europe. BVD control programmes have been classified as either systematic, involving a monitored, goal-oriented reduction in incidence and prevalence across a regional or national cattle industry, non-systematic, where measures are implemented on a herd basis as a bottom-up approach without wider systematic monitoring [21]. Three key elements for the systematic control of bovine pestiviruses have been described: the identification and elimination of congenitally persistently infected (PI) immunotolerant animals [22]; increased surveillance to monitor the progress of interventions and detect new infections [23] and measures to prevent the infection of pestivirus naïve animals and (re-) introduction of the virus into BVD-free herds. All of this might be achieved by strict biosecurity protocols, including quarantine for incoming animals, but in most cases will require the assistance of vaccines to make the control measures more sustainable, particularly in farms where biosecurity is difficult to maintain [24].

## 3. Immunity to Bovine Pestiviruses

Antibodies against pestiviruses are acquired from maternal colostrum or following an active immune response due to infection or vaccination and the importance of neutralising antibodies has been well documented [25,26]. Neutralising antibody responses have been described to target envelope glycoproteins E1 and E2, with E2 being immunodominant, playing a major role in the attachment of the virus on to a target/host cell that the neutralising antibodies inhibit [27,28]. Studies of the immune response to Pestivirus A (BVDV-1) have suggested a role for both antibody and T cell responses in protection [29,30,31,32]. The further characterisation of antibody and T cell targets that elicit protective immune responses to pestiviruses therefore remains an important prerequisite in the design of next-generation vaccines. 

There is good evidence for the role of T cell responses in BVD immunity. Calves vaccinated in the presence of maternal antibodies, whilst unable to mount an effective antibody response, do generate memory T cells sufficient to protect against subsequent viral challenge [33]. Both CD4^+^ and CD8^+^ T cell responses have been shown to be evoked by Pestivirus A (BVDV-1) [34], although the antibody depletion of CD4^+^ T cells, but not CD8^+^ or γ/δ T cells, has been shown to increase the duration of virus shedding [35]. These CD4^+^ T cell responses have been shown to be directed principally against E2 and NS3 but also to other proteins such as the N^pro^, C and E^rns^ proteins [36,37,38]. An assessment of longitudinal responses to all the different BVDV proteins in the course of natural infection has not been conducted. However, E2 and NS3 have been shown to be the immunodominant proteins by assessment of ex vivo T cell responses to peptide pools representing the whole Pestivirus A (BVDV-1) proteome following experimental infection [39]. 

## 4. Cross-Protection between Bovine Pestiviruses

Given the range of Pestivirus species that may infect cattle and cause BVD, it is evident that broadly cross-protective vaccines are required for the control of pestiviruses in cattle, buffalo and other bovid species. While good cross-protection has been observed within the wide range of Pestivirus A (BVDV-1) genotypes, the need to adapt vaccines to include atypical bovine pestiviruses (Pestiviruses H/BVDV-3) has been considered in the development of vaccines [40,41]. 

Clinical cross-protection against Pestivirus B (BVDV-2) challenge has been reported using Pestivirus A based MLV vaccines [42]. However, the inability to fully prevent foetal infection, postnatal infection and virus shedding has, in part at least, been attributed to antigenic diversity [18,43]. Accordingly, Pestivirus B has been included in some newer vaccine preparations [44,45]. The comparison of T cell and antibody responses observed after infection with a Pestivirus A and/or a Pestivirus H (BVDV-3) virus showed limited immune reactivity to Pestivirus H in Pestivirus A infected animals but good reactivity to Pestivirus A in Pestivirus H inoculated animals [46]. Highly conserved targets such as non-structural protein NS3 generated recall T cell responses in both groups, while viral glycoprotein E2 responses were virus species specific. 

Considering the proteomes of bovine pestiviruses these results are not surprising, but they allow for the rationale design of future vaccines that should convey a broad cross-protection. It has been well described that Pestivirus A can be separated into genotypes a-u [4,47], but all of these belong to the same serotype with antibodies widely cross-reacting. Indeed, the amino acid (aa) identity of Pestivirus A genotypes is >85% for the polyprotein, compared to the identity between Pestiviruses A, B and H of 71–76% (data not shown). 

The differences within and between pestiviruses are the highest in the E2 glycoprotein that mediates the viral attachment and is under selective pressure from neutralising antibodies. Here, the identity among Pestivirus A E2 proteins is 68–78% and between Pestivirus A to B is 61–66%, while between Pestivirus A and H it is only 57–63%. Accordingly, the aforementioned lack of T-cell cross-reaction between E2 of different pestiviruses and a limited ability to cross-neutralise is not surprising. More so, when we focus on the E2 aa 1-271 that are known to contain the host-cell interaction mediating domains DA, DB, and DC [48], the aa identity among Pestivirus A is further reduced to 66–76% and between the three pestivirus species A, B and H to 50–62% (Table 1). These figures give an approximation of the challenge for vaccine design, without taking discontinuous, conformational epitopes into account while conversely some of the changes might not affect the neutralising epitopes. More research is needed to precisely identify the molecular events and binding partners involved in virus attachment and fusion, as well as defining neutralising epitopes on the pestivirus envelope E2 protein. 

Conversely, NS3, the other main immunogenic protein identified, is highly conserved among Pestivirus A (>94%) and between Pestiviruses A, B and H (>89%, Table 2). Accordingly, broad cross-reactivity for the T-cells directed against NS3 can be assumed. This cross-reactivity would support both cytotoxic CD8^+^ T cell and CD4^+^ helper T cell responses and thus indirectly support B cell responses. Accordingly, it is tempting to speculate that the observed clinical cross-protection at least observed by Pestivirus A vaccines, or after infection of cattle with other pestiviruses is indeed T cell driven.

It seems feasible to consider a vaccine design that takes only a limited amount of conserved T cell reactive antigens such as NS3 and a mix of E2 (possibly only domains DA-DC) to induce neutralising antibodies into account. This, however, highlights crucial limitations of both the immune system and some of the current state-of-the-art approaches to design modern vaccines: the immune system cannot make a rational decision; it will induce B (antibody) and T cell responses to all antigens presented in a vaccine. Accordingly, traditional inactivated vaccines would likely distract the immune response into too many unnecessary and unhelpful directions. Conversely, some of the promising modern platforms that can mimic MLV will struggle with this design too. Both replication deficient and recombinant pestiviruses use the exchange of one protein (or parts of it). Such a vaccine cannot be designed carrying multiple E2 variants. Similarly, the delivery of multiple proteins is a challenge for viral vector vaccines—unless variants of these are combined in one product. DNA, RNA or subunit vaccines would have an advantage here but are not (yet) ready in their design and proof of concept for production that they can be used with same efficacy as MLV or surrogates thereof. 

## 5. Vaccination against Bovine Pestiviruses

According to VeVax, an online licensed veterinary vaccines database [49], there are more than 120 registered BVD vaccine products currently in use around the world, mostly in North and South America. These are conventional modified live virus (MLV) or inactivated/killed virus vaccines, formulated as either Pestivirus A and/or B (BVDV-1 and/or -2) preparations or multivalent vaccines including other pathogens implicated in the bovine respiratory disease complex, such as members of the Pasteurellaceae family (including *Mannheimia haemolytica*, *Pasturella multicoda* and *Haemophilus somni*), bovine herpesvirus-1, parainfluenza type 3 virus and bovine respiratory syncytial virus [50].

Vaccination against bovine pestiviruses is an additional control measure aimed at the protection of post-natal calves against infection after maternally-derived antibody wanes and of heifers to prevent foetal infections which may result in reproductive failure, foetal losses and birth of PI calves, which continuously spread the virus [51]. The adoption of BVD vaccination in Europe is voluntary with uptake ranging from 20–75% [52]. BVD eradication without vaccination has been successfully carried out by large-scale eradication schemes in Scandinavian countries where 90–99% of herds are considered free of Pestiviruses A and B (BVDV-1 and -2) [53]. These schemes are, however, expensive and intensive and take a long time to implement. The addition of vaccination remains a cost-effective measure for disease control [54]. Furthermore, in regions with high cattle densities and BVDV prevalence, and therefore an increased probability of virus reintroduction into naïve herds, vaccination can easily be incorporated into systematic control strategies [55].

### 5.1. Modified Live Virus Vaccines

MLV vaccines are more efficacious since they induce high titres of virus neutralising antibodies and provide a longer duration of protection from clinical disease than inactivated vaccines that often require booster immunizations to achieve sustained protection [56]. However, there is a risk that MLVs may revert to a virulent form or recombine with field viruses and cause disease and vaccinated animals have been reported to develop transient viremia and to shed vaccine virus [57,58]. MLV vaccines have also been shown to confer foetal protection after Pestivirus A (BVDV-1) and, in a few instances, Pestivirus B (BVDV-2) challenge following vaccination with Pestivirus A based MLV vaccines [59,60,61]. In pregnant animals, however, live vaccines pose the risk of the vertical transmission of vaccine virus that can occasionally result in foetal complications or birth of PI calves [62]. MLV vaccination has also been implicated in post-vaccination mucosal disease, when PI animals do not mount an immune response to BVDV, due to their intra-uterine infection, and a fatal condition develops, characterised by severe lesions of the oral and intestinal mucosa [63]. As a result of these safety concerns, MLVs are not licensed in all countries.

### 5.2. Inactivated Vaccines

Whilst inactivated vaccines are generally safer and therefore preferred for the vaccination of breeding cattle, bovine neonatal pancytopenia (BNP) has highlighted that there too problems may arise. BNP was a syndrome associated with an inactivated BVD vaccine that affected calves in their first month of life, characterised by pancytopenia, severe bleeding and high lethality that had originally been described in Europe. The vaccine design was rational: prepare the virus in a bovine cell line to reduce allergic or other reactions [64]. What had not been considered sufficiently was the ability of such a preparation to induce some form of auto-immune reaction as it seems to have occurred. The vaccine in question contained a significant amount of bovine (cell line derived) non-viral antigens [65] and the transfer (ingestion) of colostrum from vaccinated affected dams (i.e., those that had given birth to BNP calves) was sufficient to induce disease in several (albeit not all) calves [66]. The underlying pathogenesis of BNP is far from being fully resolved as blood transfusion to affected calves was not sufficient to overcome this problem. The nature of alloreactive antibodies associated with BNP [67] remains debatable, with bovine MHC class I molecules suggested to be the alloantigens responsible [10,68,69]. It is worth noting that BNP affected only some dams in some herds, thus an unresolved genetic component also seems to have played a role. The disease was a highly unusual event that could have been avoided through improved vaccine preparations. In principle, however, this highlights the potential for a safety problem in the use of crude inactivated BVDV preparations that contain significant amounts of host cell derived material. 

### 5.3. Differentiation of Infection from Vaccination (DIVA)

Importantly, neither MLV nor inactivated vaccines allow for DIVA [70], which reduces their suitability for use in BVD eradication efforts when vaccination and control could be monitored by Ab ELISA tests. Inactivated vaccines were previously thought to facilitate DIVA because of a lack of production of non-structural proteins such as NS3 and hence the diminished responses to these target antigens [71]. This has, however, in a number of studies proved not be the case [72,73], due to the presence of non-structural proteins in the crude virus preparations used in inactivated vaccines.

### 5.4. Sub-Unit and Next-Generation Vaccines

Recent BVD vaccine developments aim to address the shortcomings of the existing vaccines. An ideal vaccine should prevent disease, prevent vertical and horizontal virus transmission, be safe in pregnant animals, unable to revert to virulence, have broad efficacy to account for virus diversity and permit DIVA [40]. Various approaches towards the development of the next generation of BVD vaccines have been made and some already evaluated in cattle, but most still face challenges [74,75,76,77]. One such approach, DNA vaccination, has been trialled with mixed success and is generally deemed to be effective but comes with challenges such as the optimal route of delivery. The use of recombinant subunit proteins had earlier been deemed insufficient to provide effective protection, not least when focusing on E2 [39,78,79,80,81]. The combination of antigens (e.g., E2 and NS3) in formulations with molecular adjuvants to stimulate antigen-presenting cells [58,59] has been more successful, but requires further refinement. The combination of approaches such as a DNA prime, protein boost regimes [82,83,84] is overall more promising than either of the two alone, but comes with the inherent drawback of requiring at least two vaccinations. Accordingly, these types of vaccines are a challenge to the manufacturer, the veterinarian and the farmer. Vaccinations of animals in remote areas of under-developed countries are a challenge already, but additionally, farmers in developed countries and industrialised settings prefer single, possibly multivalent, or fewer multiple vaccines that require minimal inoculations. Defective viral vectored replicons [85,86,87,88,89], synthetic attenuated infectious cDNA clones [90,91], as well as chimeric pestivirus marker vaccines [92], all aim to build upon the success of MLV vaccines, but face regulatory challenges in some countries as they are genetically engineered microorganisms and those built on recombinant pestiviruses might (with regards to recombination) come with similar limitations as MLVs. 

In summary, BVD vaccine development efforts are the subject of ongoing research, following the trends of modern vaccine innovation, and have contributed to pave the way for such, including the use of RNA vaccination [70]. To further improve the design of safe and efficacious vaccines that cross-protect against several related pestiviruses and are sufficiently cheap to manufacture and apply, an improved understanding of the immunology in bovine pestivirus infections is required. 

## 6. Diagnosis of Bovine Pestiviruses

Virus isolation has historically been considered the gold standard for the detection of pestiviruses in blood and milk samples. Antigen detection by immunohistochemistry, antigen-capture ELISA and nucleic acid detection by polymerase chain reaction (PCR) based tests are now broadly applied [93,94]. The detection of bovine pestivirus-specific antibody in milk or blood samples by ELISA or by virus neutralisation test is also possible, although these tests are not able to differentiate infected from vaccinated animals (DIVA), which is a significant limitation for BVD control programmes [70]. Antibody and antigen tests, when used together, help to distinguish acutely infected from persistently infected (PI) animals. The diagnoses of both virus and immune responses can be hampered by the genetic and antigenic variability of pestiviruses. More robust approaches are required to assess antigenic relationships between the various Pestivirus species and subtypes [95]. Most tests were established against Pestiviruses A and B (BVDV-1 and -2) and have not been fully validated to detect Pestivirus H (BVDV-3) or Pestivirus D (BDV). Whilst the comparative performance of different Pestivirus A and B assays to detect antibodies against atypical Pestiviruses has been assessed [96], the existing tests are not sensitive enough and no specific tests are available. This makes the comprehensive detection of all potential bovine pestiviruses a diagnostic challenge that can be addressed by focusing on conserved parts of the genome/proteome [11,17,97].

## 7. Conclusions

Bovine pestivirus diversity, heterologous hosts and the emergence of novel ruminant pestiviruses all pose a significant challenge to control and vaccination [10]. Vaccine efficacy studies should include challenges with heterotypic bovine pestivirus species and vaccines will have to be designed with at least Pestivirus A, B and H (BVDV-1, -2 and -3) in mind. A better understanding of this diversity and consideration of vaccine correlates of protection, cross-protective potential and efficacy against various bovine Pestivirus species will improve vaccine design and thus support global BVD control to reduce the disease burden and economic impact.

## Figures and Tables

**Table 1:** E2 amino acid homology of selected Pestivirus A, B and H strains. Full genome sequences of selected strains were downloaded from GenBank; the respective accession numbers are provided. Additional information as per strain name and/or country of origin are provided in the left column. Sequences were translated into proteins where necessary and aligned with Clustal W and the sequences trimmed to contain the aa 1-271 of the E2 protein using the MacVector software package. The matrix depicts identities of amino acids (aa) above and homologies below the diagonal. The identities between the same virus species: Pestivirus A (BVDV-1), B (BVDV-2) or H (BVDV-3) are highlighted in green. The identities of Pestivirus B or H compared to Pestivirus A are highlighted in yellow.

	MH379638.1	M96687	KX987157	KR866116	LC089876	JN400273	JQ799141	AF502399	KJ000672	MH231148	KY683847	FJ040215	JQ612704
MH379638.1 BVDV-1a GB Ho916	100	75.6	73.3	73.7	71.9	69.6	66.3	58.5	56.7	60.4	54.1	52.6	53
M96687 BVDV-1b OSLOSS	84.5	100	69	72.3	72.3	69.7	64.9	57.2	56.1	59.4	52.8	52.4	52.4
KX987157 BVDV-1f SLO	82.6	79.3	100	72.2	75.2	73.7	71.1	59.6	58.1	61.5	56.7	54.4	56.7
KR866116 BVDV-1m CN	84.1	81.2	85.2	100	70.7	74.4	66.7	55.9	57.4	59.6	57.4	56.3	58.1
LC089876 BVDV-1n JP	79.6	81.2	83	81.5	100	67.4	68.5	58.5	57	58.5	55.9	55.2	54.4
JN400273 BVDV-1q SD0803 pig	82.2	81.9	84.1	83.7	78.9	100	65.9	58.1	59.3	60.7	55.2	53.3	55.9
JQ799141 BVDV-1u Yak CN	78.5	77.1	81.1	80.4	79.6	81.9	100	58.1	60.4	63	52.6	52.2	52.6
AF502399 BVDV-2a NY-93	71.9	70.5	73.3	72.2	68.1	74.4	75.2	100	80.6	80.6	52.2	54.1	51.1
KJ000672 BVDV-2b SD1301	70.4	67.5	71.1	71.5	66.3	73.7	74.1	88.4	100	81	50.7	53	50.4
MH231148 BVDV-2c 12-149150	72.2	70.5	73.3	73.3	68.1	73.7	77	90.7	87.7	100	53.7	54.4	53
KY683847 BVDV-3 BRA Bos Ind	67	67.2	71.9	71.9	71.1	70.4	67.8	65.9	65.2	66.3	100	90	94.1
FJ040215 BVDV-3 Thai KK	68.5	67.9	71.9	70.7	73	70	68.5	67.8	67	67.4	94.8	100	86.2
JQ612704 BVDV-3 IT	66.3	66.4	71.1	72.2	69.6	71.1	68.1	65.6	65.9	65.6	97	92.6	100

**Table 2:** NS3 amino acid homology of selected Pestivirus A, B and H strains. Approach and methods used are similar to Table 1. For this analysis, the full length NS3 protein sequences were used.

	MH379638.1	M96687	KX987157	KR866116	LC089876	JN400273	JQ799141	AF502399	KJ000672	MH231148	KY683847	FJ040215	JQ612704
MH379638.1 BVDV-1a GB Ho916	100	97.5	97.4	97.4	97.4	97.4	94	91.9	91.1	92.1	92.4	91.8	92.2
M96687 BVDV-1b OSLOSS	99.4	100	97.4	97.8	97.8	97.8	94.3	92.1	90.6	91.9	92.5	91.9	92.4
KX987157 BVDV-1f SLO	99.4	99.4	100	98.1	97.4	98	94.4	92.1	90.8	91.9	92.5	91.9	92.4
KR866116 BVDV-1m CN	99.4	99.4	99.4	100	97.8	98.2	94	92.7	91.4	92.5	92.4	91.5	92.2
LC089876 BVDV-1n JP	99.4	99.4	99.4	99.4	100	97.7	94	91.8	90.3	91.7	92.4	91.9	91.9
JN400273 BVDV-1q SD0803 pig	99.1	99.4	99	99.1	99.1	100	94.6	91.7	90.5	91.5	92.4	91.7	92.2
JQ799141 BVDV-1u Yak CN	98.2	98.2	98.1	98	98	98	100	90.6	89.5	89.9	91.5	91.4	91.5
AF502399 BVDV-2a NY-93	98.2	98	98.4	98.2	98	97.7	97.1	100	97.5	98.1	91.7	90.9	91.5
KJ000672 BVDV-2b SD1301	97.5	97.5	98	97.8	97.5	97.2	96.3	99	100	97.7	90.3	89.9	90.8
MH231148 BVDV-2c 12-149150	98.1	97.8	98.2	98.1	97.8	97.5	96.9	99.6	99.1	100	91.1	90.3	91.2
KY683847 BVDV-3 BRA Bos Ind	97.2	97.2	96.9	97.4	97.2	97.2	96.6	96.6	95.9	96.5	100	98.5	99.6
FJ040215 BVDV-3 Thai KK	97.2	97.2	96.9	97.2	97.2	96.9	96.6	96.3	95.9	96.2	99.3	100	98.4
JQ612704 BVDV-3 IT	97.2	97.2	96.9	97.4	96.9	97.2	96.6	96.6	96.2	96.5	99.7	99.3	100
